# PREPARING FOR ADVANCING ILLNESS AND END OF LIFE WITH DEMENTIA

**DOI:** 10.1093/geroni/igad104.1777

**Published:** 2023-12-21

**Authors:** Jenny van der Steen

## Abstract

Dementia involves coping with advancing illness that involves cognitive and functional decline. Also decision-making capacity diminishes, which implies that preparing for a future and taking opportunities to exercise individual and relational autonomy is particularly relevant in the case of dementia. Engaging the person with dementia and family caregivers meaningfully in formal or informal advance care planning conversations may require addressing specific challenges. Health care providers often lack time or are reluctant to broach these conversations. Often conversations are delayed even in institutional long-term care settings as residents, their family caregivers and care providers may all view advance care planning as uncomfortable and difficult to initiate. Special support for health care providers to develop skills, confidence and taking initiative is needed. This symposium presents about new, large qualitative and quantitative studies with contributions from the UK and Canada, and international work. It addresses preparing for an uncertain future through advance care planning and decision making about future care and treatment which may include conversations about the end of life. We present and evaluate tools to support the process across settings.

